# Hydronephrosis caused by a giant ovarian cyst

**DOI:** 10.1590/S1677-5538.IBJU.2015.0354

**Published:** 2016

**Authors:** Ha Yeon Kim, Moon Kyoung Cho, Eun Hui Bae, Soo Wan Kim, Seong Kwon Ma

**Affiliations:** 1 Department of Internal Medicine, Chonnam National University Medical School, Gwangju, Korea; 2 Department of Obstetrics and Gynecology, Chonnam National University Medical School, Gwangju, Korea

## CASE

**Figure 1:**
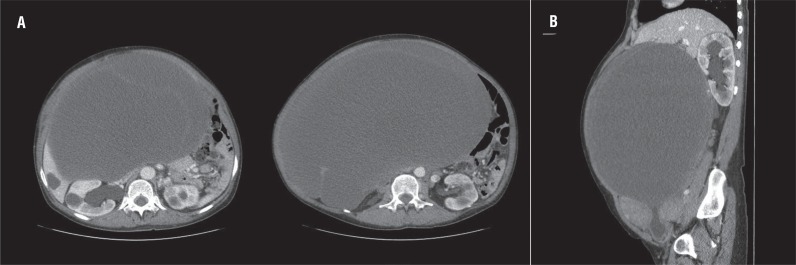
The axial (A) and sagittal scan (B) of contrast-enhanced abdominopelvic computed tomography show the hydronephrosis caused by a large cystic mass in the abdominopelvic cavity.

**Figure 2:**
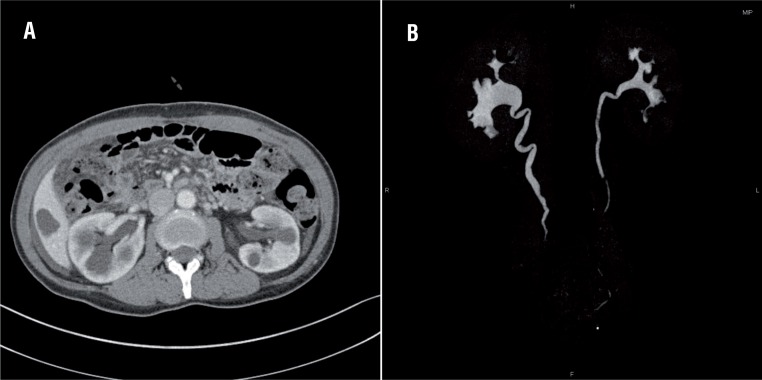
Post-operative contrast-enhanced abdominal computed tomography scan shows the partial improvement of hydronephrosis in the axial scan (A) and the coronal scan of urography (B).

A 52-year-old woman presented with abdominal distension. The laboratory data were as follows: white blood cell count, 7.100/mm^3^; hemoglobin, 12.2g/dL; platelet count, 206.000/mm^3^; blood urea nitrogen, 16.8mg/dL; serum creatinine, 1.6mg/dL; sodium, 146mEq/L; potassium, 3.2mEq/L; chloride, 94mEq/L; glucose, 110mg/dL; total protein, 8.6g/dL; and albumin, 4.9g/dL. Random urine protein concentration was 100mg/dL. Contrast-enhanced abdominopelvic computed tomography (CT) revealed a 36x21x30cm-sized cystic mass in the abdominopelvic cavity. The mass lesion had displaced adjacent visceral organs. Both kidneys were also displaced by this lesion. Bilateral hydronephrosis was observed, of which degree was more prominent in the right kidney (Figure-1). The serum level of carcinoembryonic antigen was 1.26ng/mL (reference range, 0-4.7ng/mL). The cancer antigen 125 concentration (CA-125) was 109.5IU/mL (reference range, 0-35IU/mL). An exploratory laparotomy revealed a large cystic mass originating from right ovary. The cyst contained approximately 10L of brownish fluid. Cytology of the fluid showed no evidence of malignancy. A pathological examination of the mass demonstrated a benign cystic lesion with hemorrhage and extensive thrombus formation. After surgical excision of the mass, contrast-enhanced abdominal CT urography showed partial the improvement of hydronephrosis (Figure-2).

Hydronephrosis may result from multiple diseases such as urinary tract stones, uroepithelial malignancies, anatomical abnormalities, and external compression. In woman, gynecologic diseases are important causes of hydronephrosis (1). Ovarian cysts are considered large when they are more than 5cm, and giant when they are more than 15cm (2). Giant ovarian cysts are very rare, however, when they do occur they require surgical resection because of not only the mass effect-associated morbidity and mortality but also the malignancy risk (2, 3). Furthermore, only a few cases of hydronephrosis caused by a giant ovarian cyst have been documented (2, 4). In the present case, the serum concentration of CA-125 was increased although the pathologic findings of ovarian cystic lesion and cytology of the cystic fluid were benign. The serum level of CA-125 may be increased in gynecologic malignancies. However, benign conditions also cause the increase of serum CA-125 including benign ovarian neoplasms, functional ovarian cysts, pelvic inflammatory diseases, pregnancy, menstruation (5).

In conclusion, we present a rare instructive case of hydronephrosis caused by a giant ovarian cyst. Clinicians should consider that hydronephrosis could be associated with various clinical conditions.
